# Proposed framework regarding management of patients with breast cancer and anti-cancer treatment-related elevation in cardiac troponin

**DOI:** 10.1016/j.ijcha.2024.101522

**Published:** 2024-10-17

**Authors:** Michael Cronin, Dina Neiroukh, Aoife Lowery, William Wijns, Michael Kerin, Maccon Keane, Silvie Blazkova, Osama Soliman

**Affiliations:** a University of Galway, School of Medicine, Galway, Republic of Ireland; b Netherlands: Euro Heart Foundation, Netherlands

## Abstract

Cardiac biomarkers are a vital component within the first edition of the European Society of Cardiology guidelines in Cardio-Oncology. Specifically, they are mentioned in the definition of mild asymptomatic cancer therapy-related cardiac dysfunction, where left ventricular systolic function is ≥50 % with two outcomes; either a new decrease in global longitudinal strain >15 % from baseline and/or a new rise in cardiac biomarkers above the defined 99th percentile cut off values. Cardiac troponin is one such biomarker.

Many of the treatments for breast cancer have published data on cardiac dysfunction and/or cardiovascular toxicity, and such may lead to an elevation in cardiac troponin. However, there is conflicting and incomplete data regarding how to approach an elevated cardiac troponin during anti-cancer treatment, which has confounded patient care in the clinical trial setting.

We propose a novel framework to guide physicians in treatment-related elevation of cardiac troponin in the breast cancer population. Secondly, the additive role which the recommendation that cardiac troponin carries within mild asymptomatic definitions of CTRCD is the subject of great debate. We suggest a reflection on the role of biomarkers, specifically in reference to cardiac troponin.

## Highlights

• Cardiac biomarkers are a vital component within the first edition of the European Society of Cardiology guidelines in Cardio-Oncology.
• Mild CTRCD is defined where LVEF is ≥50 % with either; a new decrease in GLS >15 % and/or a new rise in cardiac biomarkers above 99th percentile
• There is conflicting and incomplete data regarding how to approach an elevated cardiac troponin during anti-cancer treatment.
• This document proposes a novel framework to guide physicians in treatment-related elevation of cardiac troponin in the breast cancer population.
• This document addresses the role that cardiac troponin carries within mild asymptomatic definitions of CTRCD.

## 1 Introduction

Cardiac biomarkers are a vital component within the first edition of the European Society of Cardiology (ESC) guidelines in Cardio-Oncology [1]. Specifically, they are mentioned in the definition of mild asymptomatic cancer therapy-related cardiac dysfunction (CTRCD), where left ventricular systolic function is ≥50 % with two outcomes; either a new decrease in global longitudinal strain >15 % from baseline and/or a new rise in cardiac biomarkers above the defined 99th percentile cut off values. Cardiac troponin is one such biomarker. Breast cancer diagnosis and prognosis are based on a standardised approach [2], [3], [4], [5], [6], involving the ‘TNM’ model, grading, and receptor expression. Once this is established then a treatment strategy is undertaken [7], [8], defined by whether a patient has early or metastatic disease, and whether they are HER2 or oestrogen receptor-positive versus triple negative breast cancer.

Many of the treatments within these approaches have published data on cardiac dysfunction and/or cardiovascular toxicity, and such may lead to an elevation in cardiac troponin. However, there is conflicting and incomplete data regarding how to approach an elevated cardiac troponin during anti-cancer treatment, which has confounded patient care in the clinical trial setting of our institution (‘Understanding Cardiac Events in Breast Cancer (UCARE) – pilot cardio-oncology assessment and surveillance pathway for breast cancer patients’ Trial ID NCT05921279 [9]). We propose a novel framework to guide physicians in the breast cancer population. Secondly, the additive role which the recommendation that cardiac troponin carries within mild asymptomatic definitions of CTRCD is the subject of great debate. We suggest a reflection on the role of biomarkers, specifically in reference to cardiac troponin, in this short communication, drawing on recommendations from various cardiology and oncology societies (American College of Cardiology, American Society of Clinical Oncology, European Society for Medical Oncology, and American Heart Association).

## 2 Cardiac troponin

### 2.1 Background

Cardiac troponins are predominantly found within the contractile apparatus of cardiac myocytes, and both troponin I and T are irreversibly released secondary to cell death [10]. They are one of the biomarkers commonly suggested for patient observation before and during treatment with specific anti-cancer agents, predominantly HER2 therapies, anthracyclines, and immune checkpoint inhibitors. Both Troponin I and Troponin T are highly sensitive and specific for cardiac damage [11] and can be deployed as a high-sensitivity assay [12], detecting much lower serum troponin levels compared to standard assays. The term myocardial injury should be used when there is evidence of elevated cardiac troponin values with at least one value above the 99th percentile upper reference limit. This myocardial injury is considered acute if there is a rise and/or fall of cardiac troponin values [13]. Beyond their role as a standard of care in acute coronary syndrome [14], an elevation of cardiac troponin within the non-acute coronary syndrome population infers an elevated cardiovascular, and cerebrovascular, risk [15], [11]. Multiple commercial and research assays [16] are differentiated by their limit of definition, use of different epitopes/antibodies, and detection tag. A caveat remains regarding an overlap in the elevation of cardiac troponin T in the neuromuscular disease population, which does not extend to troponin I [17], and further in the yet unestablished 99th percentile cut-off in troponin assays [18] across differing sex and age populations.

### 2.2 Impact

The proposed patient benefit in the cardio-oncology population from the use of cardiac troponin assays is early detection of subclinical myocardial injury and prognostic value regarding the risk of adverse cardiovascular events [19]. In the breast cancer population there are standard systemic anti-cancer therapies used which are accepted to carry a risk of cardiac dysfunction, or cardiovascular toxicity; namely anthracyclines, immune checkpoint inhibitors, taxanes, fluoropyrimidines, cyclophosphamide, carboplatin, HER2-specific tyrosine kinase inhibitors, and nucleoside analogues [20], [21], [22], [23], [24], [25], [26], [27]. The breast cancer population are also frequently treated with targeted therapy [28], radiotherapy [29], [30], and endocrine therapy [31] where there is published data regarding their effect on cardiovascular health.

How best to use cardiac troponin assays to interpret these effects on cardiovascular health remains a question [32], and further when there is an abnormality in cardiac troponin in those on multiple systemic anti-cancer agents, establishing the causative agent remains a clinical challenge [33]. Some agents, such as trastuzumab [34], [35], [36], [37], [38] have published conflicting data on the relationship between abnormal cardiac troponin during treatment and cardiac events. The same extends to anthracyclines, where some data [39], [40] fails to correlate the elevation of cardiac troponin secondary to therapy with cardiac events, when alternate data does [41].

Recent data has demonstrated variation in the outcome of CTRCD between sex-neutral troponin versus sex-specific, where the use of sex-specific troponin led to a much higher point prevalence (10 % against 5 %) of mild CTRCD at the end of anthracycline treatment for breast cancer [42]. In this context, the upper limit of the normal reference range for sex-neutral cardiac troponin I and T was higher than that of sex-specific assays. Elsewhere, in a pooled population of high-dose chemotherapy regimes, the pattern of troponin I elevation predicted cardiovascular events at three years [43]. A *meta*-analysis [44] described a higher prevalence of left ventricular systolic dysfunction in patients who experience an elevation in troponin during treatment. This effect was described in cardiac troponin-I [45] and cardiac troponin-T [46], but interestingly, it did not carry a linear relationship between the absolute value of the troponin elevation and the degree of cancer therapy-related cardiac dysfunction. Even though this relationship is well-described, at five years in low-risk cardiovascular patients with breast cancer [40], an elevation in troponin did not result in major adverse cardiac events. This is highly significant when addressing their role within cardio-oncology societal guidelines, especially in those patients with mild asymptomatic CTRCD. In recent times, some European national societies have produced consensus documents with the aim to better emphasize the pathophysiological and clinical relevance of the evaluation over time of circulating levels of high sensitivity cardiac troponin I/T in the general population as well as in patients with some specific clinical conditions, also including the evaluation of cardiac damage associated with the use of cardiotoxic drugs [47]. Specifically, these societies within in the consensus documents suggest not varying between use of troponin I and T over the cycle of patient care, using the same testing laboratory as possible, having a pre-therapy measurement, using sex-specific values for the 99th upper limit of normal, and considering a reference change value between two consecutive troponin measurements >30 % to be significant.

#### 2.2.1 Contemporary management

Current societal guidance suggests grouping patients undergoing a specific anti-cancer therapy with CTRCD into those with and without clinical symptoms and signs of heart failure. At this juncture, the patients are separated by definition of clinical severity, and a multidisciplinary team discussion is undertaken regarding the best and safest approach for the patient. Some anti-cancer therapies (HER2 therapies, anthracycline, immune checkpoint inhibitors) have algorithmic approaches [48] that have been externally validated [49]. However this algorithmic approach does not extend to other anti-cancer treatments used in breast cancer, e.g. taxanes, fluoropyrimidines, platinum agents, cyclophosphamide, nucleoside analogues and cyclin-dependent kinase 4/6 inhibitors. Nor does it include radiotherapy or endocrine therapies.

The approach recommended [1] for these anti-cancer agents includes observation of cardiac troponin in conjunction with cardiac imaging. In most jurisdictions, this cardiac imaging is echocardiography, with nuclear medicine, magnetic resonance imaging, and computed tomography are also suitable options given local practice/available and in appropriate clinical scenarios. Given the variability in anti-cancer treatments and cardiac diagnostics used, it is imperative that the multidisciplinary team includes senior clinicians trained in the use of these treatments and diagnostics and that a case-by-case basis is employed.

In current practice, whether or not the patient is managed as an outpatient or an inpatient largely depends upon this multidisciplinary team discussion. However, in extreme examples, this may be self-evident. Heart failure therapy is a general term used within the ESC guidelines for patient management [1]. This therapy can include pharmacology and non-pharmacology measures, such as cardiac implantable electronic device therapy, electrophysiology procedures, rehabilitation courses, and coronary intervention. High-level evidence [50], [51], [52], [53], [54], [55], [56], [57], [58], [59], [60] for pharmacology use in CTRCD predominantly reference ACE inhibitors/ARB (enalapril, candesartan, perindopril, lisinopril), Beta-blockers (carvedilol, metoprolol, bisoprolol) and mineralocorticoid receptor antagonists (spironolactone). A systematic review found only a 2 % absolute difference in left ventricular systolic function when beta-blockade and renin-angiotensin system inhibitors are prescribed for CTRCD due to trastuzumab or anthracyclines [61]. Dosing and method of anti-cancer treatment delivery can also be altered as felt appropriate by the anti-cancer team.

### 2.3 Proposed framework

**Fig. 1:**
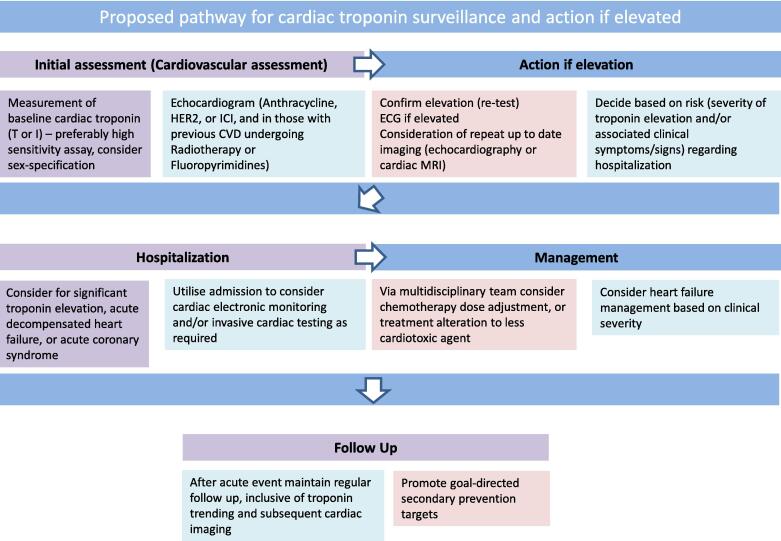
Proposed pathway for cardiac troponin surveillance and action if elevated.

In summary, we believe the available literature does not describe a reproducible approach to elevation in cardiac troponin during anti-cancer treatment for breast cancer. Standardisation of troponin assay recommendation, and consideration of sex-specific assay use, should be included in future guidelines. In this context, we propose a dedicated pathway (see Fig. 1) to guide physicians in managing troponin elevation during anti-cancer treatment. Specifically this approach initially involves an initial assessment of the patient at baseline, then action if a cardiac troponin is elevated, factors to consider regarding potential hospitalization of a patient, acute management and goals at follow-up.

Secondly, the current role of cardiac troponin in CTRCD is suboptimal. Further definitions are needed regarding troponin criteria use. Its clinical value for mild asymptomatic CTRCD is not concrete when event rates of meaningful clinical endpoints remain low, and may lead to temporary cessation of necessary anti-cancer treatment. Its role in the commencement of pharmacology in this context should also be reconsidered. ESC guidelines now confer a 2b recommendation to the commencement of ACE inhibitor/angiotensin receptor blocker and beta-blockade for mild asymptomatic CTRCD, despite a very modest difference in left ventricular systolic function in the entire population with ventricular dysfunction. We suggest that the primary role of cardiac troponin to be re-described as additional to the clinical scenario rather than central.

## 3 Conclusions

We propose a framework for the management of cardiac troponin and subsequent elevation noted during systemic anti-cancer therapy for breast cancer. Moreover, the role of cardiac troponin needs further definition in future cardio-oncology societal guidelines. Future perspectives might investigate recommended assay use, and a reflection on the benefit of cardiac troponin within mild asymptomatic cancer therapy-related cardiac dysfunction definitions.

## CRediT authorship contribution statement

**Michael Cronin:** Writing – original draft. **Dina Neiroukh:** Validation. **Aoife Lowery:** Validation. **William Wijns:** Validation. **Michael Kerin:** Writing – original draft. **Maccon Keane:** Validation. **Silvie Blazkova:** Validation. **Osama Soliman:** Conceptualization, Supervision, Revision.

## Declaration of competing interest

The authors declare that they have no known competing financial interests or personal relationships that could have appeared to influence the work reported in this paper.
